# Temporal Leadership and Bootlegging Behavior of Employees: The Mediating Effect of Self-Efficacy

**DOI:** 10.3389/fpsyg.2021.633261

**Published:** 2021-09-27

**Authors:** Mingze Li, Huili Ye

**Affiliations:** School of Management, Wuhan University of Technology, Wuhan, China

**Keywords:** temporal leadership, self-efficacy, perceived team-efficacy, bootlegging behavior, social cognition theory

## Abstract

As an important source of innovation, bootlegging is widespread in organizations. However, a lack of understanding exists in its antecedents. Based on the social cognition theory, this study aims to explore when and how temporal leadership (TL) leads to bootlegging behaviors (BOs) of employees, with self-efficacy (SE) as a mediator and perceived team efficacy (TE) as a moderator. We conducted a two-stage questionnaire survey and collected data from 231 employees from four companies located in Wuhan, P.R. China. SPSS and Mplus are used for testing our model, and the results are shown as following: TL positively affects the BO of employees. Besides, SE plays a mediating role in the relationship between TL and bootlegging, and perceived TE has a moderating effect between TL and SE. Also, perceived TE moderated the indirect effect of TL on bootlegging via SE. This study identifies the internal mechanism between time management and bootlegging, which provides an instructive view for further study on organizational innovation management. Theoretical contrition and practical implication have been discussed in this study.

## Introduction

Innovation is important during the development of an organization. If an organization wants to gain advantages in fierce competition, it needs to innovate constantly (D'aveni, 2010). In fact, many innovations do not begin formally in organizations but during private activities by employees (Knight, 1967; Augsdorfer, 2008). That is, bootlegging behavior (BO) refers to the spontaneous and secret innovation behavior of employees which is expected to be beneficial to an organization (Criscuolo et al., 2014). This kind of behavior is common in the technology industry and manufacturing industry. In these industries, there is a great demand for innovation, but formal innovation is sometimes with many restrictions on the implementation, and many innovative products come from BO. For example, BMW's 12-cylinder engine and Agilent Technologies' 1200 series chemical test equipment were the products of BOs by employees. Having made these products marketable, the companies gained a competitive advantage over their competitors (Masoudnia and Szwejczewski, 2012). An investigation showed that 5–10% of employees in an innovation team have BO (Augsdorfer, 2005), of which 70.9% of them will lead to product and process innovation (Masoudnia and Szwejczewski, 2012). 3M has a 15% rule that encourages employees to bypass management and engage in private innovation in the hope that employees will bring beneficial innovations to the company. Since this kind of behavior is a prevalent and important phenomenon in the organization, there are still questions needed to be answered.

One such question is why and when individuals will conduct BO. Although not only innovation comes from formal activities which are permitted by their leader and organized with the specific innovation projects (Kwon and Kim, 2020) but also it can emerge from some informal innovative activities, like BO (Augsdorfer, 2005). While scholars put their sights more on the antecedent of the formal innovation activities, such as the creative behavior of employees (Shafi et al., 2020; Chen et al., 2021), then they ignored the informal innovation activities, such as BO. They pointed out that BO can also facilitate organizational innovation (Iyer and Davenport, 2008; Masoudnia and Szwejczewski, 2012). For example, Criscuolo et al. (2014) found that the bootlegging activity of an individual will help get a high level of innovative performance. Zhao et al. (2020) also found that BO can facilitate work engagement of employees and then increase their innovative performance. We knew less about the antecedence of those informal innovation activities, as well as an examination and discussion of its influencing factors and internal mechanisms (Wang et al., 2019), which is a limitation for understanding the source for innovation. Few studies explored the antecedence of BO, for example, based on strain theory, Globocnik and Salomo (2015) suggested that formal management practices can promote the BO of employees. Criscuolo et al. (2014) also found that the autonomy of an individual at work and organizational accountability can influence the effort employee paid in bootlegging activities. However, previous studies focused on the individual and organization policy factors in influencing BO, lacking attention to leadership. Based on the theory of planned behavior, Jia et al. (2021) started to explore the factors in leadership, and his study suggested that paradoxical leadership can facilitate the emergency of BO of employees in China context. Leadership plays an important role in the work attitude and behavior of employees, and we needed more theoretical frameworks to understand the relationship between leadership and BO.

This study focuses on leadership about time management, and we proposed that temporal leadership (TL) can facilitate BO of employees. TL emphasizes time reminders, time planning, time scheduling, and time resource allocation (Shamir, 2011; Mohammed and Harrison, 2013). In the innovation-required business environment, time management is an important part of organizational management. Facing a business environment full of rapid product renewals, managers need to pay increasing attention to time management. TL often sets task deadlines for employees, tracks the progress of tasks of team members, and properly distributes time resources. These behaviors are called TL (Ancona et al., 2001). These tracking methods may reduce employee autonomy (Gagné and Deci, 2005) and cause employees to focus too much on their own tasks and less to no time on innovate, which may limit their innovative behaviors (Oldham and Cummings, 1996; Amabile et al., 2002). Surprisingly, some empirical studies have suggested that TL can effectively promote the innovations of employees (Zhang et al., 2021). Since TL may limit innovations of employees, it may promote the formation of team innovation. As such, one must ask whether team innovation promoted by TL comes more from the emergence of BO of employees? Based on the above suggestion, this study focuses on BO and discusses the relationship and internal mechanisms between TL and BO.

**Figure 1:**
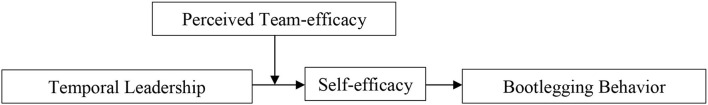
Theoretical model.

According to the social cognition theory, self-efficacy (SE) is one of the key cognitions of an individual to the environment and an important factor affecting the behaviors of individuals (Bandura, 1986; Wood and Bandura, 1989). TL creates clearer work goals for employees by arranging, synchronizing, and allocating time resources (such as spare time), which makes employees more focused on their work (Hubens, 2011). These clearer goals allow employees to acquire more skills and information related to work and thus, have a stronger ability and confidence in the control of work tasks and consequences (i.e., a sense of SE) (Llorens et al., 2007). Only when an individual believes that his/her behavior can achieve the expected effect, he/she can carry out such behavior (Bandura, 2010). As BO carried a certain probability of risk (Masoudnia and Szwejczewski, 2012), it is only when employees believe that they have a high probability of success in the BO that they will invest extra resources and time into it. Therefore, we proposed that SE plays a mediating role between TL and BO. We also proposed that perceived team efficacy (TE) of employees plays an important moderating role when employees have an insufficient perception of the ability of the team, and a positive leadership style will become important energy to support SE of employees. Therefore, when the perceived TE of employees is lower, then TL, as a supportive leadership style, is more likely to promote SE. Therefore, we proposed perceived TE as a moderator of the relationship between TL and SE. The research model for this study is shown in Figure 1.

To sum up, based on the social cognition theory, we will explore the influence of TL on BO and test the mediating effect of SE on the relationship between them and the moderating effect of perceived TE in the first stage. The research questions of this study are as follows:

RQ1: Does TL positively affect the BO of employees?

RQ2: How does SE intervene between TL and BO of employees?

RQ3: How does the perceived TE of employees play a substitute effect between TL and SE of employees?

We aimed to expand the prior studies in the following ways. First, in this study, TL and BO are connected for the first time, and the relationship between them is discussed. Previous studies have focused on the outcome of innovation behavior, and less attention has been paid to BO, which is an important source of organizational innovation (Criscuolo et al., 2014). Using TL as a starting point, this study discusses how to promote BOs of employees, by not only revealing the antecedent of BO but also enriching our understanding of team innovation. Second, based on the social cognition theory, this study explores the mediating effect of SE at the individual level, revealing the internal trigger mechanism of the relationship between TL and BO. Third, from the perspective of the psychological perception factors of employees, this study explores the moderating role of perceived TE between TL and SE and responds to the call of scholars to explore the boundary mechanism of TL.

This study is structured as follows: the “Theory and Hypotheses” section develops the hypotheses of this study, discussing the relationship among TL, SE, perceived TE, and BO of employees. The “Methods” section of this study discusses about sample, procedure, and measures. The “Analyses and Results” section discusses about analyses and results. The “Discussion” section presents the theoretical contribution and practical implication of this study and points out the limitations and future research. The “Conclusion” section gives a brief summary of the conclusion of this study.

## Theory and Hypotheses

### TL and SE

According to the social cognition theory, the external environment is an important factor that affects the behavior of an individual (Bandura, 1991); the behavior of a leader in an organization is an important external environment for employees (Cheung and Wong, 2011; Saleem, 2015). TL refers to how leaders act and think in time dimensions under the context of interactions with subordinates. The original definition of TL focused on team TL, emphasizing the influence and role of team TL in the team. For example, some studies focused on team TL coordinate the diversity of time rhythms of team members to maximize team performance (Mohammed and Nadkarni, 2011), how team TL can relieve team time pressure to improve team performance (Maruping et al., 2015), and how to solve team time conflict of team members (Santos et al., 2016). Mohammed and Alipour (2014) also emphasized the role of TL in the dyadic context. Recent studies have also begun to pay attention to individual TL (Xiao et al., 2020). For example, Op't Hoog (2009) defined individual TL as the leader behavior of identifying time preferences of employees within the time constraints and complex environments, and then, leaders can conduct differentiated management leadership behavior about time. TL in the dyadic context emphasizes how leaders perform and think from the time dimension in the context of the interaction with employees. This study focuses more on the interaction between employees and their leaders. Specifically, TL in this study is defined as a leadership style in which leaders help subordinates carry out tasks most effectively through their understanding of time and complex tasks (Op't Hoog, 2009). TL emphasizes core management activities about time as follows: time scheduling, time correspondence, and time resource allocation (Mohammed and Alipour, 2014). When leaders involve their teams in technology and competition cycles and conduct management activities across multiple time frames, they implement TL (Ancona et al., 2001).

Drawing on social cognition theory, we suggested that TL can positively affect the SE of employees. Social cognitive theory points out that mastery experiences, modeling, social persuasion, and physiological states will develop a sense of efficacy of people (Wood and Bandura, 1989). First, TL can help increase the mastery experiences of employees. TL helps employees plan their task cycles and coordinates the rhythm of task completion, which improves their concentration on the task (Hubens, 2011). Thus, employees will be more involved in task-related activities. This involvement will enable them to grasp more task-related information, which will be helpful with their mastery experience and improve their SE (Schunk, 1995).

Second, TL helps set a model for their employees, which is one of the sources of SE of employees. The study by Shakill (2019) has shown that employees will be inspired by their leader who is with TL, that is, TL increases the identification with the leader. When leaders practice the TL, i.e., setting and reminding the due dates, they show their mastery and control of this group. Eden (1992) also has suggested that leadership will have a model effect for employees, enhance SE of employees, and then increase their performance.

Third, TL is a supportive leadership style, which will give “realistic encouragements” (Wood and Bandura, 1989, p. 365) to their employees (Mohammed and Nadkarni, 2011). TL will help increase the beliefs of the capabilities of employees. In the process of helping employees coordinate their time resources, employees may perceive the support of the leader and turn work pressure into motivation factors (Maruping et al., 2015). This shift in thinking is conducive to the generation of SE (Benight and Bandura, 2004). Besides, TL also helps the teams communicate and coordinate within a complex time frame. It also helps employees deal with time-related issues, such as improving time-based communications and internal team interaction processes (Gevers et al., 2006), and promote positive work experiences (Kerns, 2012), which are the catalysts for beliefs of capabilities of employees.

Based on the above information, this study proposes the following hypothesis:

Hypothesis 1: TL is positively associated with SE.

### SE and BO

According to the social cognitive theory, SE is related to the extent to which they are able to carry out their actions in dealing with future situations (Bandura, 1982). SE refers to a belief that the ability of an individual can achieve the expected behavioral effects (Spenner et al., 1986; Chen et al., 2004). Previous studies have confirmed the positive influence of SE on the innovation activities of employees. For example, in his theory of individual creative action, Ford (1996) proposed that competency beliefs, similar to SE, can help individuals to carry out creative activities. Ahlin et al. (2014) conducted a survey on 14 million American enterprises and showed that SE of an entrepreneur is beneficial to the product innovation of enterprises and the process innovation by enterprises.

Bootlegging behavior refers to the spontaneous and secret innovation behavior of employees which is expected to be beneficial to an organization (Criscuolo et al., 2014). Even though the original intention of BOs of employees was to benefit their organizations (Augsdorfer, 2005), BOs of employees are often not supposed by official resources, which makes their completion more difficult compared with “transparent innovation,” which is supported by leaders (Augsdorfer, 2008). In this study, we distinguished BO from innovative deviances (Mainemelis, 2010; Acharya and Taylor, 2012). Carried out secretly or openly by employees, innovative deviance is an innovative behavior that leaders have explicitly forbidden, while BOs are not generally denied or forbidden by leaders as their original intentions are to benefit organizations (Augsdorfer, 2005).

Individuals with high SE are more confident about their innovational results and more likely to carry out BOs. Specifically, social cognitive theory suggests that SE can enhance the beliefs of employees in regard to overcoming obstacles (Wood and Bandura, 1989). They will regard difficult tasks as challenges to overcome and work hard to cope with them (Bandura, 2010). As mentioned above, BO is more difficult to process than innovation behavior. With SE, employees will be more confident with the success of their creative idea, even though there is a lack of support from an organization. Previous research gave evidence that SE positively affected the engagement of challenging tasks (Park and John, 2014).

Employees with high SE are more likely to “visualize success scenarios that provide positive guides for performance” (Wood and Bandura, 1989, p. 366), as such, they are more willing to pay more attention to how to successfully implement goals (Van den Broeck et al., 2010). Social cognitive theory suggests that a positive future vision can foster the persistence of their behavior (Lent et al., 1984). They do not give up quickly about what they want to do (Locke et al., 1984). This belief makes employees more likely to pursue and stick to their determination in order to complete particular ideas. In addition, they are less likely to pass up any innovation opportunities.

The internal motivation logic of SE reflects the possible driving effect of SE on BO. Employees with high SE have more internal motivation (Prabhu et al., 2008), which is an important factor to promote individual innovation (Deci and Ryan, 1987). Internally motivated individuals work more due to their own wills and choices, rather than out of a goal to get a reward or avoid guilt. They pay more attention to information outside their task, which helps them perform additional innovative activities for developing their bootlegging ideas (Judge et al., 2005).

Previous studies also suggested that SE may be a predictor for the emergence of bootlegging (Globocnik and Salomo, 2015). We thus proposed that employees with high SE are more likely to hide novel ideas which may not be implemented at present based on the principles of the organization. Informal innovation continues underground: BO. The employees may wait until the idea is more likely to be implemented before bringing it up with their managers. As such, the following hypothesis is stated:

Hypothesis 2: SE is positively associated with BO.

Combining Hypothesis 1 and Hypothesis 2, we proposed the indirect effects of TL on BO via SE. TL provides time-based task guidance to employees, which can stimulate their concentration on tasks, thus obtaining more information from the task and enhancing task-related knowledge and skills. At the same time, TL can also give employees a sense of leadership support, which is conducive to the enhancement of the SE of employees. When SE of employees is increased, their adherence to their innovative ideas will be promoted (Locke et al., 1984). Besides, it will encourage employees to have a stronger motivation to carry out additional innovative behaviors (Judge et al., 2005). Thus, the following hypothesis is formalized:

Hypothesis 3: SE mediates the relationship between TL and BO.

### The Substitute Effect of Perceived TE

The social cognitive theory posits that perceived TE, which refers to perceptions of employees of the overall ability of a team to accomplish tasks (Feltz and Lirgg, 1998), is derived from individual evaluations of achievements of a team (Bandura, 1977). The perceptions of SE of people and their expectations of the results change with their perceptions of TE (Bandura, 2002). Although teams that have performance or higher levels of innovation will have higher perceived TE (Bandura, 2002), it does not mean that perceived TE is equal to the objective ability or performance of the team of employees. Perceived TE comes from the comparison between own teams of employees and other teams, and it is a subjective feeling from the focus employee about the ability of his/her teams to accomplish tasks. When team members believe that their teams have higher achievements than other teams, their perceived TE will be higher, which is not equal to the absolute value of the achievements of teams (Bandura, 1990). Therefore, the level of perceived TE may affect the relationship between TL and SE.

Drawing on social cognitive theory, TL can effectively stimulate the SE of employees, especially when employees perceive TE is lower. TL is conducive to the integration of team task resources, which allows for the establishment of team cooperation and strengthens interpersonal relationships (Santos et al., 2016; Najam et al., 2018). When employees perceive TE is lower, TL helps employees to gain the initiative to improve the achievements of their team, which promotes SE. Besides, they may become concerned about the ability of their team to solve organizational tasks. In such a situation, the implementation of TL could coordinate and allocate time resources of employees, making employees more aware of their roles in the team and thus, enhancing their SE (Stetz et al., 2006). In contrast, when perceived TE is higher, the predictor role of TL on SE maybe not necessary. Yin et al. (2017) extended the substitutes for leadership theory (Kerr and Jermier, 1978) and pointed out that the perception of employees and evaluation of the team will affect the impact of leadership, and when employees hold a strong positive attitude toward their team, the power of the leadership on the behavior of employees will be reduced. So, when the perceived TE of employees is higher, they can get a stronger belief that their team has enough ability to achieve team goals, which gives them the confidence to achieve their own task goals (Bandura, 2002). The impact of leadership on their own efficacy will not be necessary. Thus, we proposed that, only when the employee perceived TE is at a lower level, the positive impact of TL on the SE of employees can be enhanced. Therefore, the following hypothesis is proposed:

Hypothesis 4: Perceived TE has a substitute effect between TL and perceived TE when predicting BO. Specifically, when perceived TE is lower, the relationship between TL and SE is stronger, while when perceived TE is higher, TL is not necessary for predicting BO.

Considering the relationship between SE and BO, this study proposes that when perceived TE is higher, TL cannot promote SE, and therefore, employees lack the belief in themselves to complete their own bootlegging ideas. When perceived TE is lower, TL will have a stronger promoting effect on the SE of employees and, as such, will stimulate more BOs. Therefore, this study proposes the following hypothesis:

Hypothesis 5: Perceived TE moderates the indirect effect of TL on BO through SE. Specifically, when perceived TE is lower, the indirect effect of TL on BO through SE is stronger.

## Methods

### Sample and Procedure

To test our hypotheses, we spent 3 months conducting a questionnaire at four companies in Wuhan, Hubei Province, China. After our preliminary investigation, we noticed that the TL phenomenon is widespread in these four companies, and the leaders in these four companies often conduct practical TL management of the team. In addition, these four companies have great demand for innovation. So, we collected data from these companies. We adopted the method of employee self-evaluation to inquire about TL, SE, perceived TE, and BO. First, we contacted directors from various companies to explain the purpose and significance of the research. Once the directors approved this study, we matched leaders to their employees according to the personnel structure of the company. Then, with the help of the company leaders, we issued questionnaires to the participants, introduced the research contents, promised that the data would only be used for research purposes, and promised that the data would stay private. We distributed 420 questionnaires containing demographic information and TL questions. Of these questionnaires, 350 were returned. Once we eliminated the questionnaires containing missing data, we had a sample of 335 questionnaires remaining. A month and a half later after the first round of questionnaires was returned, we distributed the second round to the same participants. These questionnaires focused on SE, perceived TE, and BO. Of the 335 questionnaires distributed, 231 with valid data were returned. The sample breakdown is as follows: 108 males (46.8%) and 123 females (53.2%), 155 (67.4%) participants between 19- and 30-year-old and 76 (32.6%) over 30-year-old, 127 people (55.0%) had a bachelor's degree or above, and 84.3% had at least 1 year of work experience.

### Measures

Our participants were all Chinese, so following the approach of Rasool et al. (2019), we first generated items from key literature, and then we conducted translation and back translation. A five-point Likert scale was used for the answers, which ranged from 1 (strongly disagree) to 5 (strongly agree). A comprehensive research questionnaire is provided in the Appendix. The study and questions in these scales did not involve any potential risks for participants.

#### Temporal Leadership

Temporal leadership was evaluated by the employees and measured using a seven-item TL scale (Mohammed and Nadkarni, 2011). We used this scale to measure the extent that supervisors practice TL. Sample items included “My direct leader usually reminds me of important deadlines” and “My direct leader usually prioritize tasks and allocate time to each task.” The Cronbach's alpha coefficient for the TL scale was 0.910.

#### SE

SE was evaluated by the employees and measured using an eight-item SE scale (Chen et al., 2001). We used this scale to measure the extent of the beliefs of capabilities of employees. Sample items included “I will be able to achieve most of the goals that I have set for myself,” and “When facing difficult tasks, I am certain that I will accomplish them.” The Cronbach's alpha for the SE scale was 0.943.

#### Perceived TE

Perceived TE was evaluated by the employees and measured using an eight-item SE scale (Chen et al., 2001). We used this scale to measure the extent of beliefs of employees of capabilities of their team. We changed “I” to “our team” as in “Our team will be able to achieve most of the goals that it has set for itself” and “When facing difficult tasks, I am certain that our team will accomplish them.” The Cronbach's alpha for the perceived TE scale was 0.951.

#### Bootlegging Behavior

Bootlegging behavior was evaluated by the employees and measured using a five-item bootlegging scale (Criscuolo et al., 2014). We used this scale to measure the extent of spontaneous and secret innovation behavior of employees which is expected to be beneficial to an organization. Sample items included “I proactively take time to work on unofficial projects to seed future official projects,” and “I am running several pet projects that allow me to learn about new areas.” The Cronbach's alpha scale for BO was 0.739.

#### Control Variables

In this study, gender, age, education, and tenure were selected as the control variables as the previous research suggested that those factors could influence the emergency of BO of an employee (Globocnik and Salomo, 2015). We also controlled the relationship tenure as previous research pointed out that it can influence the perception of the leadership with employees (Robert and Wilbanks, 2012). Considering the nested data structure, age, gender, and education level of the leader were also controlled in this study (Asparouhov and Muthen, 2008).

### Analytical Strategy

Mplus version 7.0 (Muthén B and Muthén, 1998–2018) and (SPSS 24.0; SPSS Inc., Chicago, IL, USA) were used for the analysis. First, we conducted a confirmatory factor analysis and average variance extracted (AVE) to confirm the validity among the measurement. Second, the Harman's single-factor test method was conducted to avoid having common method biases. Third, we provided descriptive statistics and correlation analysis of these variables. Finally, we performed the hypothesis testing, and a path analysis was used in this part to test our theoretical model.

In the hypothesis testing, maximum likelihood estimation with robust standard errors (MLR) is used in this study, and we set the “analysis: TYPE=TWOLEVEL” due to the nested data structure (employee responses within teams). Mplus version 7.0 was used to perform this multilevel model (Muthén B and Muthén, 1998–2018). The open-source R software (Selig and Preacher, 2008) (http://www.quantpsy.org/medmc/medmc.htm) was used to test the 95% CIs based on the Monte Carlo method. To test the mediating effect of SE, we performed the multilevel model using Mplus version 7.0 and calculated the CIs of this indirect effect. To test the moderating effect, we first centered TL and perceived TE and then created an interaction variable. Mplus 7.0 and open-source R software helped with performing the mediated moderation model.

## Analyses and Results

### Validity of the Constructs

In this study, Mplus version 7.0 was used for confirmatory factor analysis to evaluate the discriminant validity between TL, perceived TE, SE, and BO. As shown in Table 1, the four-factor model had better fitting effect, and the indicators were fitting in an ideal range (Chi-Square over Degrees of Freedom χ^2^/df = 2.317, Comparative Fit Index (CFI) = 0.905, Tucker-Lewis Index (TLI) = 0.895, Standardized Root Mean Square Residual (SRMR) = 0.065, and Root Mean Square Error of Approximation (RMSEA) = 0.076), while the remaining four index model failed to fit the standard. Therefore, TL, perceived TE, SE, and BO have good discriminative validity.

**Table 1:** Fitting indexes of different factor models.

**Model**	**χ^**2**^**	***df***	**χ^**2**^/ *df***	**CFI**	**TLI**	**SRMR**	**RMSEA**
Four-factor model (TL; SE; TE; BL)	797.167	344	2.317	0.905	0.895	0.065	0.076
Three-factor model (TL+SE; TE;BL)	1665.610	247	6.743	0.723	0.698	0.135	0.128
Two-factor model (TE+SE; TL +BL)	1843.261	349	5.282	0.686	0.660	0.121	0.136
One-factor model (TL +TE+SE+BL)	2630.905	350	7.517	0.520	0.482	0.149	0.168

*TL, temporal leadership; TE, perceived team-efficacy; SE, self-efficacy; BL, bootlegging behavior*.

We also tested the convergent validity of all constructs, and AVE and composite reliability (CR) were reported in Table 2. All constructs had met the general requirements of CR (≥0.7) (Fornell and Larcker, 1981). The measurement of TL, SE, and perceived TE had met the general requirements of AVE (≥0.5) (Fornell and Larcker, 1981), and the measurement of BO had an acceptable standard of AVE (Fornell and Larcker, 1981; Lam, 2012).

**Table 2:** Descriptive statistics and correlations (*N* = 231).

	**Mean**	**SD**	**1**	**2**	**3**	**4**	**5**	**6**	**7**	**8**	**9**
***Between***											
1. Leader gender	0.387	0.489	1.00								
2. Leader age	34.839	9.125	−0.153	1.00							
3. Leader education	3.581	0.925	0.002	0.378^**^	1.00						
***Within***											
1.Gender	0.535	0.500	1.00								
2.Age	29.574	7.519	−0.118	1.00							
3. Education	3.550	0.865	0.051	0.236^***^	1.00						
4. Tenure	5.544	7.294	0.009	0.839^***^	0.029	1.00					
5. Relationship tenure	2.963	3.016	0.078	0.483^***^	0.159^*^	0.579^***^	1.00				
6. Temporal leadership	3.863	0.609	0.044	0.010	0.051	0.04	−0.034	0.630 (0.922)			
7. Self-efficacy	3.750	0.577	0.019	0.185^**^	0.161^*^	0.180^**^	0.146^*^	0.239^***^	0.626 (0.930)		
8. Bootlegging behavior	3.247	0.560	−0.084	0.173^**^	0.086	0.151^*^	0.107	0.148^*^	0.349^***^	0.480(0.817)	
9. Perceived team-efficacy	3.905	0.639	0.034	0.168^*^	0.165^*^	0.148^*^	0.196^**^	0.279^***^	0.604^***^	0.228^**^	0.657 (0.939)

* *represents p < 0.05*,

** *represents p < 0.01, and*

*** *represents p < 0.001*.

*CR and AVE are shown in the diagonal of the table. The outside of the brackets is the result for AVE, and the inside is the result for CR*.

### Common Method Biases

To reduce the concern of common method biases, the Harman single-factor method was used in this study (Podsakoff et al., 2003; Jakobsen and Jensen, 2015). The results of factor analysis showed that five factors were extracted, and the cumulative variance interpretation percentage of the first factor was 37.541%, which did not exceed 40%. Unmeasured latent marker construct (ULMC) (controlling for the effects of an unmeasured latent method factor) was also used to test the CMB, and by following the suggestions from the study of Podsakoff et al. (2003), we added a method factor to our original four-factor model, and the results show that the model fit did not gain a great improvement (ΔCFI = 0.008, ΔTLI = 0.005, ΔRMSEA = 0.002) (Chen et al., 2016). Besides, the results of model fit for the one-factor model which is shown in Table 1 was not good yet. Overall, it could be considered that there were no common method bias concerns in this study.

### Descriptive Statistics and Correlation Analysis

Table 1 shows the mean, SD, and correlation coefficient of each variable. The correlation coefficient of each variable does not exceed 0.75, indicating that there is no collinearity problem among major variables. As can be seen from Table 3, TL and SE showed a significant positive correlation (*r* = 0.239, *p* < 0.001), SE and BO showed a significant positive correlation (*r* = 0.349, *p* < 0.001), and TL and BO showed a significant positive correlation (*r* = 0.148, *p* < 0.04), which provided preliminary support for the hypothesis of this study.

**Table 3:** Regression analysis results (*N* = 231).

**Variable**	**Self-efficacy**		**Bootlegging behavior**	
	**Model 1**	**Model 2**	**Model 3**	**Model 4**
***Within level***				
Gender	0.025	−0.021	−0.089	−0.091
Age	0.011	−0.001	0.008	0.008
Education	0.054	0.041	−0.003	0.008
Tenure	−0.002	0.008	−0.002	−0.003
Relationship tenure	0.016	−0.005	0.010	0.010
Temporal leadership	0.109^**^	0.031	0.051	0.050
Perceived team-efficacy		0.306^***^	−0.013	
Temporal leadership ^*^ perceived team-efficacy		−0.080^**^	0.037	
Self-efficacy			0.301^***^	0.288^**^
*R* ^2^	0.259	0.195	0.234	0.234

* *represents p < 0.05*,

** *represents p < 0.01, and*

*** *represents p < 0.001*.

### Hypothesis Testing

In this study, the Monte Carlo method was used to test the mediating effect and the moderated mediating effect. Mplus version 7.0 and open-source R software were used to test the hypothesis. The Monte Carlo bootstrapping cases were set to 20,000. The gender, age, education, tenure, and working years of leaders were added into the model as within-level control variables, and gender, age, and education level of leaders were added into the model as between-level variables. Hypothesis 1 proposed that TL positively affects SE. As shown in Model 1 in Table 3, TL can significantly positively predict SE (*b* = 0.109, *p* < 0.01), so hypothesis 1 was supported. Hypothesis 2 proposed that SE positively affects BO. As shown in Model 4 in Table 3, SE can significantly positively predict BO (*b* = 0.288, *p* < 0.001), so hypothesis 2 was supported. Hypothesis 3 proposed that SE plays a mediating role in the relationship between TL and BO; the Monte Carlo method was used to test the mediation effect of SE; as shown in Table 4, the indirect effect did not contain zero (*b* = 0.031, *p* < 0.05, 95% CI [0.010, 0.067]); and since SE mediated the relationship between TL and BO, hypothesis 3 was supported.

**Table 4:** Indirect and conditional indirect effect test (*N* = 231).

		**Estamate**	**SE**	**95% CI**	
				**BootLLCL**	**BootULCL**
Mediation		0.031^*^	0.015	0.010	0.067
Perceived team-efficacy	M+1SD	−0.006	0.010	−0.027	0.017
	M−1SD	0.025^*^	0.013	0.004	0.057
	Diff	−0.031^*^	0.013	−0.060	−0.010

* *represents p < 0.05*.

**Figure 2:**
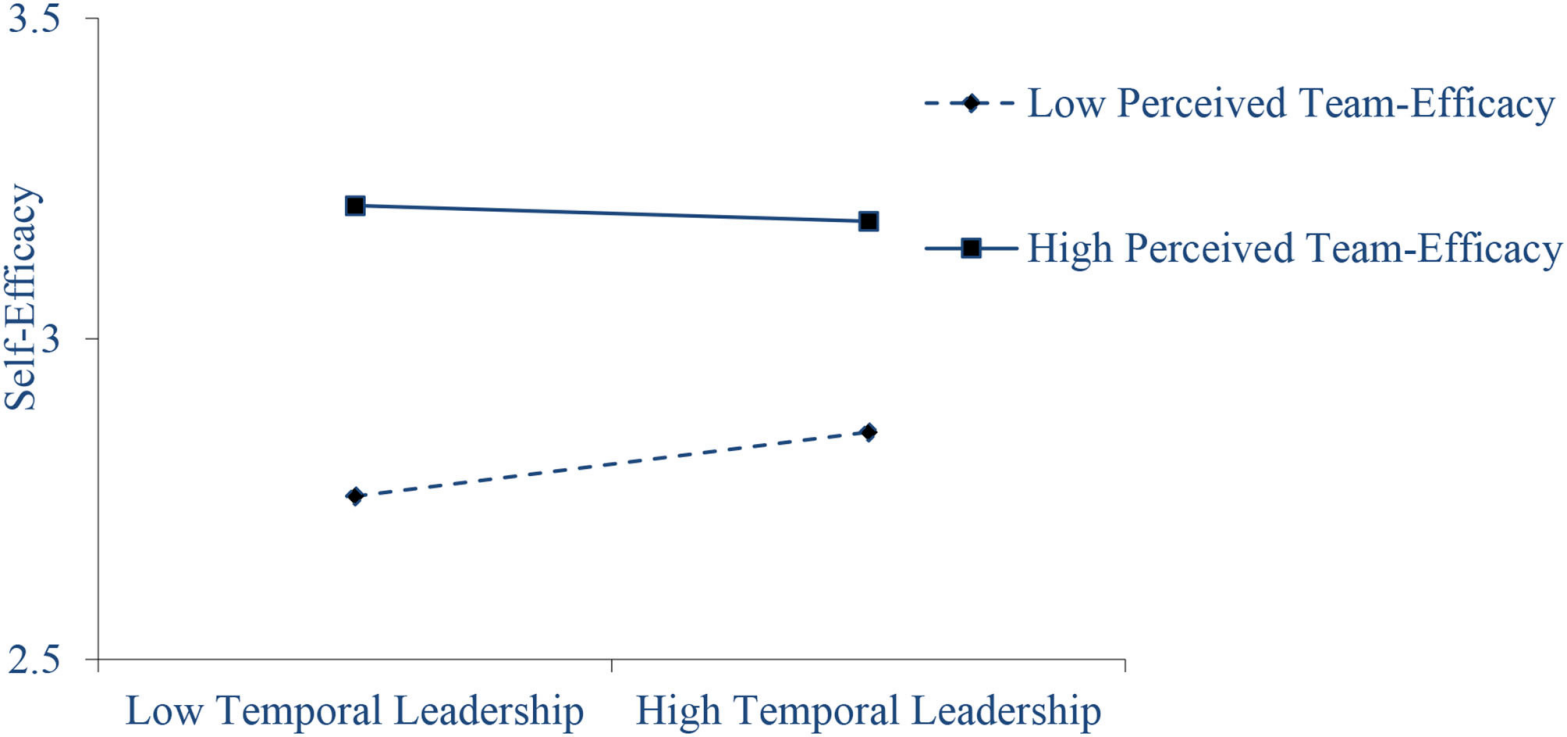
The moderating effect of perceived team-efficacy on the relationship between self-efficacy and bootlegging behavior.

In this study, the moderating effect of perceived TE was tested. First, the independent variable and the moderator were centered, and the interaction of the two variables was made. As shown in Table 3, the interaction of TL and perceived TE can significantly predict SE (*b* = −0.080, *p* < 0.01). We also conducted a simple slope test to help to understand the moderating effect, and the results show that when perceived TE was low (M−1SD), the regression slope of TL in predicting SE was −0.082, *p* < 0.05, while when perceived TE was high (M+1SD), the regression slope of TL in predicting SE was −0.020, *p* > 0.05. The difference in these two slopes were significant (*b* = −0.102, *p* < 0.01), with a 95% CI of [−0.177, −0.026], excluding 0. This indicates that perceived TE moderates the relationship between TL and SE. To clearly show the regulating effect of perceived TE, this study drew the regulating effect diagram as shown in Figure 2. Figure 2 shows that when perceived TE is lower, TL has a stronger promoting effect on SE. In conclusion, hypothesis 4 was supported.

Hypothesis 5 proposed a moderated mediation model. Similarly, the Monte Carlo method was used to test the model, and the bootstrapping cases were set to 20,000 and used in this study to test this model. As shown in Table 4, the indirect effect was significant when perceived TE is lower (M−1SD; *b* = 0.025, *p* < 0.05, 95% CI [0.004, 0.057]). When perceived team efficiency is higher, the indirect effect was not significant (M+1SD; *b* = −0. 006, *p* > 0.05, 95% CI [−0.027, 0.017]). The difference in the conditional indirect effect was significant (*b* = −0.031, *p* < 0.05, 95% CI [−0.060, −0.010]). Hypothesis 5 was supported.

## Discussion

### Theoretical Contributions

This study makes the following contributions to the research on leadership and innovation. First, this study extends our understanding of the antecedents of BO of employees. Although previous studies suggested that BO can benefit from innovation performance (Criscuolo et al., 2014), an increasing number of studies have been conducted on the antecedents of formal innovations (Gong et al., 2009; Zhang and Bartol, 2010), and there was less effort paid for knowing how to facilitate the informal innovation such as BO. Only limited studies have explored the individual and organizational policy factors, such as formal management practices (Globocnik and Salomo, 2015), and autonomy of an individual (Criscuolo et al., 2014), while scholars pay less attention to the leadership factors which are also important for promoting work behavior of employees (Jia et al., 2021). This study links TL with BO, discusses its internal mechanism, and reveals that TL can positively affect BO of an employee, which is helpful in exploding the antecedent of BOs of employees from a time perspective. This perspective is important because we are facing a more competitive business environment, and it is needed to pay effort to get interaction between the TL and BO to know how the BOs are facilitated by leadership on the time context.

Second, we contributed to the social cognition theory by exploring the trigger mechanism of BO and examined the mediating role of SE in the relationship between TL and BO. Previous studies explore the mechanism from the strain theory and the theory of planned behavior (Globocnik and Salomo, 2015; Jia et al., 2021), and this study got a new insight into the mechanism to understand why TL can promote BO of employees. Although TL creates an “urgent” work environment for employees (Gevers and Demerouti, 2013) and reduces their autonomy (Hubens, 2011), this study finds that TL makes employees focus more on the task time and increases employee confidence in engaging in informal innovation activities, which drives BO of employees via their SE, which shows that the innovation promoted by TL can exist with the form of BO. This study solves a seemingly contradictory problem in previous literature: although TL reduces the autonomy of employees (Mohammed and Alipour, 2014), it is beneficial to the innovation of employees (Zhang et al., 2021), which may be with the form of BO.

Finally, using the substitutes for leadership theory (Kerr and Jermier,1978), we proposed a moderated mediation model and tested the substitute effect of perceived TE. Previous literature had not tested the boundary conditions of the emergence of BO (Criscuolo et al., 2014; Globocnik and Salomo, 2015; Jia et al., 2021). This study tests the important factor of perceived TE of employees, which is conducive to enhancing the understanding of the relationship between TL and BO, and also provides reference and guidance for managers. Specifically, The perceived TE of employees was an important factor that affected attitudes and behaviors of employees related to innovation. As such, this study shows that when perceived TE of employees was lower, TL in regard to promoting individual SE played a more effective role, while when perceived TE of employees was higher, the effect of TL on individual SE was substituted by perceived TE of employees.

### Practical Implications

This study provides guidance for organizational management practices from the following aspects. First, this study proves that TL has a positive effect on employee BO. Although TL limits the autonomy of employees to some extent, it also makes employees more willing to spend extra resources by increasing their job involvement and SE (i.e., BO).

Second, this study finds that effective time management by leaders is beneficial to organizational innovation. As such, we encouraged managers to allocate time resources reasonably in a team, reminded employees of task deadlines, and assisted employees in time planning and other behavioral assistance, so as to help employees deal with time-complex tasks, which can generate positive work attitudes and behaviors.

Third, the trigger mechanism (i.e., SE) of BO identified in this study can be generalized. In addition to the TL implemented by managers, other management behaviors in an organization that can cause SE should be considered, such as organizational support, high-quality leader-member relationships, skill training, and person-post matching (O'Driscoll and Randall, 1999). R&D companies with high innovation needs can arrange appropriate positions based on the expertise of their employees, actively carry out technical training for employees, and provide them with more support, so as to enhance their senses of SE and stimulate their BOs.

Fourth, this study finds that perceived team effectiveness also played an important role in moderating the relationship between TL and BO. When the perceived TE of employees was lower, the effectiveness of TL in promoting personal efficacy was stronger. Therefore, in the process of organizational innovation management, managers should pay attention to the perceived TE of employees. When perception of team efficacy of employees is lower, they should implement TL to enhance SE of employees, thus triggering better work attitudes and behaviors. For example, managers can enhance communication with employees to understand their true thoughts about the team. If employees feel that a team is inefficient, then managers should proactively take measures to intervene in the behaviors of employees.

### Limitations and Future Research

This study also has some limitations. First, this study explores the mediating effect of SE based on the social cognition theory, while there are many other possible mechanisms in the relationship between leadership and BO. For example, motivation also plays an important role in the relationship between organizational context and BO of employees. Besides, a resource perspective may be a useful way to understand the emergence of BO of employees. More studies can be done to generate a more complete map for the bootlegging literature.

Second, this study examines only the moderating effect of perceived TE, focusing on the influence of perceived TE between leaders and employees on the relationship between TL and SE. As such, there are other possible moderators. For example, compared with employees with the prevention focus, employees with the promotion focus may be more confident and more likely to undertake BOs because they are less sensitive to losses and pursue goals more aggressively (Higgins, 1997). In addition, an organizational innovation atmosphere may be beneficial to the relationship among TL, SE, and BO because teams with the higher innovation atmosphere are more tolerant and approve the special operation modes of innovations of employees (Scott and Bruce, 1994), which are conducive to reducing the psychological burden of BO of employees. More boundary conditions should be explored in future research.

Third, our research encourages the management to facilitate the BO via TL. Although lots of research and practice provided the evidence that BOs have a positive impact on innovative objectives (Augsdorfer, 2005, 2008; Globocnik and Salomo, 2015), it is noted that bootlegging may cause a major violation of the rules of the organization (Criscuolo et al., 2014). For example, employees who engage in BO will spend less time in their in-role tasks and cost more materials of the organization (Augsdorfer, 2005). Even employees with too much expectation for the success of his/her bootlegging project can cause a deeper disappointment when the bootlegging project is rejected by their supervisor. Considering the possible dark side of BO, Hooi and Tan (2021) gave an insight into the coping strategy in fostering positive outcomes of BO, and future research can explore how can we get more benefit from BO and how can we reduce the possible dark side of BO.

Finally, the data in this study are self-evaluation data of employees. Although efforts were made within this study to reduce concerns of common method biases, the data were collected at different times, and a Harman single factor test was used to examine the extent of common method biases. The risk still exists. Besides, our data were within the team, so we did not control the item variance at the team level by using the Harman single factor test. Therefore, further research can use other methods, such as text analysis or experimental design, to examine the relationship between TL and BO to obtain more robust conclusions.

## Conclusion

Based on the social cognition theory, this study explores the relationship between TL and BO of employees. Specifically, this study reveals that TL has a positive impact on the BO of employees, while there is a mediating role of SE in the relationship between TL and BO. Besides, perceived TE plays a moderating role in the relationship between TL and SE, and a moderated mediation model is also supported.

This study is concluded as follows: first, TL has a positive effect on facilitating SE of an employee, and this kind of leadership can build up the belief of capability of employees through management activities of the leader about time, namely, time scheduling, time correspondence, and time resource allocation. This result helps to expand the impact of TL on the innovation of employees. Second, according to the social cognition theory, this study verifies that TL has an indirect effect on BO via SE. In this research, it is believed that the SE of employees is an important factor related to BO. TL can be a predictor to BO of employees by increasing belief of capability of an employee, which filled the gap for the lack of antecedent of BO. Finally, perceived TE has a substitute effect between TL and perceived TE when predicting BO. Specifically, when perceived TE is lower, the relationship between TL and SE is stronger, while when perceived TE is higher, TL is not necessary for predicting BO. Thus, management can practice TL to their team members when they find a low perception of TE in their team members.

## Data Availability Statement

The raw data supporting the conclusions of this article will be made available by the authors, without undue reservation.

## Ethics Statement

Ethical review and approval was not required for the study on human participants in accordance with the local legislation and institutional requirements. Written informed consent for participation was not required for this study in accordance with the national legislation and the institutional requirements.

## Author Contributions

All authors listed have made a substantial, direct and intellectual contribution to the work, and approved it for publication.

## Funding

This study was supported by the Ministry of education of Humanities and Social Science project (18YJC630073), the Natural Sciences Foundation of China (71802154), the Fundamental Research Funds for the Central Universities (2020VI033; 2020-ZY-061), and the China Postdoctoral Science Foundation (2019M662734).

## Conflict of Interest

The authors declare that the research was conducted in the absence of any commercial or financial relationships that could be construed as a potential conflict of interest.

## Publisher's Note

All claims expressed in this article are solely those of the authors and do not necessarily represent those of their affiliated organizations, or those of the publisher, the editors and the reviewers. Any product that may be evaluated in this article, or claim that may be made by its manufacturer, is not guaranteed or endorsed by the publisher.

## Appendix

**Table:**

**Constructs**	**Items**
Temporal leadership	My direct leader reminds me of important deadlines
	My direct leader prioritizes tasks and allocate time to each task
	My direct leader prepares and build in time for contingencies, problems, and emerging issues
	My direct leader paces my work process so that work is finished
	My direct leader urges me to finish subtasks on time
	My direct leader sets milestones to measure progress on the project.
	My direct leader is effective in coordinating the team to meet client deadlines
Self-efficacy	I will be able to achieve most of the goals that I have set for myself.
	When facing difficult tasks, I am certain that I will accomplish them.
	In general, I think that I can obtain outcomes that are important to me.
	I believe I can succeed at most any endeavor to which I set my mind.
	I will be able to successfully overcome many challenges.
	I am confident that I can perform effectively on many different tasks.
	Compared to other people, I can do most tasks very well.
	Even when things are tough, I can perform quite well.
Perceived team-efficacy	Our team will be able to achieve most of the goals that it has set for itself.
	When facing difficult tasks, I am certain that our team will accomplish them.
	In general, I think that our team can obtain outcomes that are important to the team.
	I believe our team can succeed at most any endeavor to which it set for itself.
	Our team will be able to successfully overcome many challenges.
	I am confident that our team can perform effectively on many different tasks.
	Compared to other teams, our team can do most tasks very well.
	Even when things are tough, our team can perform quite well.
Bootlegging behavior	I have the flexibility to work my way around my official work plan, digging into new potentially valuable business opportunities
	My work plan does not allow me the time to work on anything other than the projects I have been assigned to.
	I enjoy tinkering around with ideas that are outside the main projects I work on
	I am running several pet projects that allow me to learn about new areas.
	I proactively take time to work on unofficial projects to seed future official projects.
